# Genetic variations in IKZF3, LET7‐a2, and CDKN2B‐AS1: Exploring associations with metabolic syndrome susceptibility and clinical manifestations

**DOI:** 10.1002/jcla.24999

**Published:** 2024-01-09

**Authors:** Alireza Paniri, Mohammad Mahdi Hosseini, Sadegh Fattahi, Galia Amiribozorgi, Mohsen Asouri, Mansooreh Maadi, Nima Motamed, Farhad Zamani, Haleh Akhavan‐Niaki

**Affiliations:** ^1^ Genetics Department, Faculty of Medicine Babol University of Medical Sciences Babol Iran; ^2^ Zoonoses Research Center Pasteur Institute of Iran Amol Iran; ^3^ Faculty of Medicine Babol University of Medical Sciences Babol Iran; ^4^ Gastrointestinal and Liver Diseases Research Center, Iran University of Medical Sciences Tehran Iran; ^5^ Department of Social Medicine Zanjan University of Medical Sciences Zanjan Iran

**Keywords:** LncRNAs, metabolic syndrome, microRNAs, non‐coding RNAs, obesity, polymorphism, SNPs

## Abstract

**Background and aim:**

Metabolic syndrome (MetS) increases the risk of atherosclerosis and diabetes, but there are no approved predictive markers. This study assessed the role of specific genetic variations in MetS susceptibility and their impact on clinical manifestations.

**Method:**

In this study, a genotype–phenotype assessment was performed for IKZF3 (rs907091), microRNA‐let‐7a‐2 (rs1143770), and lncRNA‐CDKN2B‐AS1 (rs1333045).

**Results:**

Analyses indicate that while rs907091 and rs1143770 may have potential associations with MetS susceptibility and an increased risk of atherosclerosis and diabetes, there is an observed trend suggesting that the rs1333045 CC genotype may be associated with a decreased risk of MetS. The genotypes and allele frequencies of rs1333045 were significantly different between studied groups (OR = 0.56, 95% CI 0.38–0.81, *p* = 0.002, and OR = 0.71, 95% CI 0.55–0.92, *p* = 0.008), with the CC genotype displaying increased levels of HDL. Furthermore, the rs907091 TT genotype was associated with increased triglyceride, cholesterol, and HOMA index in MetS patients. Subjects with the CC genotype for rs1143770 had higher HbA1c and BMI. In silico analyses illustrated that rs907091 C remarkably influences the secondary structure and the target site of a broad spectrum of microRNAs, especially hsa‐miR‐4497. Moreover, rs1333045 creates a binding site for seven different microRNAs.

**Conclusion:**

Further studies on other populations may help confirm these SNPs as useful predictive markers in assessing the MetS risk.

## Graphical abstract

The mechanism of genetic polymorphisms involved in the regulation of the obesity process, blood pressure, blood sugar levels, and lipid metabolism in metabolic syndrome development. *IKZF3* rs907091 is associated with increased levels of TG, cholesterol, insulin, and HOMA, raising the risk of MetS susceptibility. Moreover, the rs1333045 CC is associated with the increased HDL levels compared to the TC + TT genotype and thereby decreases the risk of MetS. The results of the present study showed the association between CC and CT genotypes of LET7‐a‐2 rs1143770C>T with increased levels of HbA1C, BMI, and CRP, and probably higher risk of MetS susceptibility.

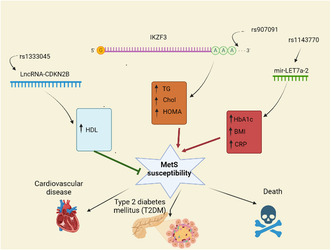

## 1 INTRODUCTION

Metabolic syndrome (MetS), also known as syndrome X and insulin resistance syndrome, is a multifactorial disorder that is considered a major risk factor for cardiovascular disease, type 2 diabetes mellitus (T2DM), stroke, and cardiovascular mortality.^1^, ^2^, ^3^ It is worth mentioning that there are different risk factors including insulin resistance, obesity, atherogenic dyslipidemia (hypertriglyceridemia and reduced levels of high‐density lipoprotein cholesterol (HDL‐C)), and hypertension implicated in MetS development.^4^, ^5^ Apart from that, other factors such as age, higher body mass index (BMI) and HbA1c level, elevated diastolic blood pressure (DBP) and waist circumstance, elevated low‐density lipoprotein (LDL) and triglyceride (TG), decreased HDL, and alcohol consumption could raise the susceptibility to MetS progression.^6^, ^7^, ^8^ Despite a large number of studies on the mechanism, diagnosis, and therapeutic strategies, MetS remains a main health concern worldwide.^9^, ^10^, ^11^ The global prevalence of MetS has been estimated to be about 20%–25% and would be higher in the Asian population (12%–37%).^7^, ^12^ Therefore, identifying highly specific biomarkers to predict susceptibility to MetS might help prevent disease development and even death.

In the Iranian population, nitric oxide synthase 3 (*NOS3*)‐c.894G>T was found to be associated with an increased MetS risk in Iranian–Azerbaijanis, with the BMI influencing the effects of *NOS3*‐c.894G>T genotypes on MetS risk.^13^ Another study investigated the interactions between rs1761667 polymorphism and dietary patterns on cardiometabolic risk factors and MetS risk in apparently healthy individuals. Adherence to a diet rich in fiber, fish, and dairy products demonstrated a more pronounced effect on cardiometabolic risk factors in A‐allele carriers compared to the GG genotype of rs1761667 polymorphism.^14^ Additionally, kernel machine regression models in the Tehran Cardio‐metabolic Genetics Study assessed the association between BUD13 homolog (*BUD13*), ZPR1 zinc finger (*ZPR1*), and apolipoprotein A‐V (*APOA5*) SNPs in the 11p23.3 region with lipid‐related traits in MetS, especially those affected by high triglyceride levels.^15^

Correspondingly, recognizing non‐coding RNAs (ncRNAs) involved in the regulation of lipid metabolism, insulin secretion, and blood pressure control might pave this road. Several studies illustrated that IKAROS Family Zinc Finger 3 (*IKZF3*), long non‐coding RNAs cyclin‐dependent kinase inhibitor 2B (lncRNA *CDKN2B‐AS1*), and microRNA lethal 7‐a2 (*let7‐a‐2*) are involved in lipid metabolism disorders, type 2 diabetes mellitus, diabetic neuropathy, inflammation, cerebral and cardiovascular diseases, and atherosclerosis disorders through regulating numerous pathways comprising B lymphocyte proliferation and differentiation and lipid (especially TG and cholesterol) metabolism.^16^, ^17^, ^18^, ^19^ Given the wide spectrum functions of the above genes, any variation, particularly single‐nucleotide polymorphisms (SNPs), in their sequence might dramatically influence their expression and function and could consequently promote MetS development.

The present study aimed to determine the association between *IKZF3* rs907091 T>C, microRNA*‐LET7A2* rs1143770 C>T, and lncRNA*‐CDKN2B‐AS1* rs1333045 T>C and the risk of its development. To the best of our knowledge, this is the first study investigating the association of these SNPs with MetS.

## 2 MATERIALS AND METHODS

### 2.1 Subjects

This study included 369 (252 men and 117 women) patients with MetS and 200 (137 men and 63 women) normal subjects. The subjects were diagnosed and recruited at 17 Shahrivar hospital in Amol, Iran, based on a cohort study which is ongoing since 2008.^20^, ^21^ The subjects were screened and selected according to the following inclusion and exclusion criteria: the inclusion criteria consisted of age > 20 years; TG > 150 mg/dL, HDL (female < 50 mg/dL, male < 40 mg/dL), waist circumference (female > 89 cm, male > 102 cm), fasting blood sugar (FBS) >100 mg/dL,^22^ and mean arterial pressure (MAP) >100 mmHg,^23^ and the exclusion criteria consisted of subjects with underlying diseases and infection with Epstein–Barr virus (EBV) or cytomegalovirus (CMV). Biochemical criteria (TG, cholesterol, LDL, HDL, aspartate aminotransferase [AST], alanine aminotransferase [ALT], HbA1C, FBS, gamma‐glutamyl transpeptidase [GGT], C‐reactive protein [CRP] and alkaline phosphatase [ALP]), hematological index (hemoglobin [Hb], mean corpuscular volume [MCV], mean corpuscular hemoglobin [MCH], hematocrit [HCT], and mean cell hemoglobin concentration [MCHC]), and other clinical and physiological parameters including systolic blood pressure (SBP), DBP, MAP, height, weight, BMI, waist, hip, and fatty liver were collected from existing records. All participants provided written consent before they participated, and all protocols were approved by the Ethics Committee of Babol University of Medical Sciences (IR.MUBABOL.REC.1400.039).

### 2.2 Single‐nucleotide polymorphism selection

SNPs including *IKZF3* rs907091 T>C (3′UTRs variant), microRNA‐*LET7A2* rs1143770 C>T (promoter variant), and lncRNA *CDKN2B‐AS1* rs1333045 T>C (intronic variant) were retrieved from the SNP databases of ENSEMBL v70, NCBI db SNP, and HapMap by considering minimum minor allele frequency (MAF) ≥ 0.05.

### 2.3 DNA extraction and genotyping

Genomic DNA was isolated from peripheral whole blood by the salting‐out method. Genotyping of IKZF3, microRNA‐LET7‐a‐2, and lncRNA‐CDKN2B genes was performed using PCR‐RFLP. The PCR primers were designed using AlleleID 7.0 (Table 1). The PCR and single‐base extension were performed in a 20‐μL reaction mixture, containing 1.5 μL genomic DNA (20–50 ng), 1 μL of each primer (0.05 μmol), 8.5 μL H_2_O, and 8 μL PCR master mix (Pars tous, Iran). PCR reactions were carried out using a thermocycler (Eppendorf, Hamburg, Germany), under the following conditions: 95°C for 5 min followed by 35 cycles of 94°C for 20 s, annealing temperature for 35 s (Table 1) and then 72°C for 35 s, and a final extension step of 72°C for 5 min.

**TABLE 1:** The sequence of primers and PCR‐RFLP conditions.

Polymorphisms	Primer sequences (5′ → 3′)	Allele	Region	Annealing temperature (°C)	Amplicon size (bp)	Enzyme	Fragment length (bp)
rs907091	F: GGTGAGGCTTCCCAGCAAGGTC	T/C	3′UTR	62	371	NciI	C = 245, 126 T = 371
	R: GACCTCGCCTAACAGATTGCTCTC						
rs1143770	F: GAAGGGAGCAAATACTTGGGACTG	C/T	Promoter	62	429	HincII	C = 285, 110 T = 414
	R: GAGAAATGCTGAAACAGTCAACTCTG						
rs1333045	F: CATTGGAGTCAGGCTGCTGGAG	T/C	Intron	62	470	MvaI	C = 190, 280 T = 470
	R: GACCTCGCCTAACAGATTGCTCTC						

### 2.4 In silico analysis

Bioinformatics online databases including PolymiRTS Database 3.0^24^ and miRNASNP v2.0^25^ were recruited to investigate microRNA binding sites. In addition, the RNAhybrid v2.1.2 tool was engaged in order to analysis the impact of SNPs on putative microRNAs targets. In this regard, highly stable microRNA/mRNA duplexes show a very low minimum free energy (MFE) (kcal/mol), and MFE was calculated for both variant alleles. Moreover, the Sfold 2.2 web server was used to predict the impact of different alleles of SNPs on the secondary structure.

### 2.5 Statistical analyses

The sample size for this genetic association study was determined through power analysis using the QUANTO program.^26^ Setting the significance level (*α*) at 5% and aiming for a statistical power (1 − *β*) of 80%, we conducted the analysis based on the prevalence of MetS in the Iranian population, which was derived from the literature and established at 0.26.^27^ Additionally, we considered the MAF of the selected SNPs, obtaining this information from the PubMed SNP database, with a specific focus on frequencies observed in the other Asian population. Notably, the selected sample size was calculated to meet the smallest sample size suitable for each of the three SNPs, further enhancing the reliability and robustness of our investigation.

Clinical characteristics and demographic data of studied subjects and their genotypes were statistically analyzed using R programming language (version 4.0.3)^28^ given that the *Q*–*Q* plot and Shapiro–Wilk test showed that some of the continuous variables do not follow a normal distribution, median, interquartile range (IQR) and Mann–Whitney *U* test were considered instead of mean, standard deviation, and Student's *t*‐test, respectively. Furthermore, the Chi‐square test and linear regression model were adjusted with confounders including sex, age, height, CRP, hemoglobin, ALT, AST, ALP, GGT, MCV, MCH, MCHC, and smoking history. Moreover, the odds ratio (OR) and its corresponding 95% confidence interval (95% CI) were calculated. Three central body obesity indices including BMI (Weight/Height^2^), Waist‐to‐Hip Ratio (WHR), and Waist‐to‐Height Ratio (WHtR) also were included in the features. Pearson's Chi‐square test was analyzed for each polymorphism in controls to find out any possible deviation from Hardy–Weinberg equilibrium (HWE) and genotyping error. While genetic polymorphisms are more likely to function in only one inherited way, we aimed to throughly investigate the potential associations between the genetic polymorphisms and MetS by considering multiple genetic models (co‐dominant, dominant, overdominant, recessive, and log‐additive), providing a more comprehensive understanding of their role. Genetic models and genotype associations analyses were conducted applying the R package SNPassoc.^29^ This package allows incorporating the specified confounders as covariates in the logistic regression model. By considering these confounders in our analysis, we aimed to minimize their potential influence on the observed associations between the genetic polymorphisms and the outcome of interest, such as MetS. The false discovery rate was controlled by Benjamini–Hochberg (BH) correction, and an adjusted *p*‐value <0.05 has been considered statistically significant.

## 3 RESULTS

### 3.1 Patients' characteristics

The study groups included 369 MetS patients and 200 normal subjects with 68% male participants in each group (Table 2). The mean age of MetS patients and healthy controls was 50.44 ± 14.65 years and 49.92 ± 15.23 years, respectively. Results showed significantly higher values of fatty liver (grade I–III), diabetes, waist, weight, BMI, hip circumference, WHR, FBS, TG, GGT, ALT, HbA1c, SBP, DBP, MAP, WHtR, homeostatic model assessment for insulin resistance (HOMA) index (*p* < 0.0001), CRP (*p* < 0.008) and serum insulin levels (*p* < 0.013), and lower values of HDL (*p* < 0.0001) in patients compared to the control group. It is worth noting that there were no significant differences between age, height, cholesterol, Hb, MCV, MCH, HCT, MCHC, LDL, ALP, and AST in the studied groups.

**TABLE 2:** Demographic characteristics of metabolic syndrome patients and controls.

Clinical characteristics	Cases (*n* = 369)	Controls (*n* = 200)	*p*‐value	
	Mean ± SD	Mean ± SD	BH	
	Median (IQR)	Median (IQR)		
Sex				
Male	252	137	1	
Female	117	63		
Fatty				
Non‐fatty	173	132		>0.0001*
Grade I	94	38	0.5127	
Grade II	74	21		
Grade III	28	9		
Diabetes				
Diabetic	114	18	<0.0001*	
Non‐diabetic	255	182		
Age	50.44 ± 14.65	49.92 ± 15.23	0.69	
Waist circumference (cm)	93.57 ± 9.52	87.5 ± 10.87	<0.0001*	
Height (cm)	165.11 ± 9.61	165 ± 10.3	0.94	
Weight (kg)	81.56 ± 14.9	74.23 ± 15.64	<0.0001*	
Hip (cm)	103 (98–108)	99 (93–106)	<0.0001*	
FBS (mg/dL)	110 (103–129)	95 (90–102)	<0.0001*	
TG (mg/dL)	140 (95–195)	99 (79–133)	<0.0001*	
Cholesterol (mg/dL)	185.28 ± 41.72	184.08 ± 43.05	0.65	
GGT (U/L)	26.7 (21.55–36.9)	22.1 (17.8–30.8)	<0.0001*	
CRP (mg/dL)	1 (1–4)	1 (0–2)	0.013*	
Hb (g/dL)	14.4 (13.2–15.57)	14.25 (13–15.3)	0.15	
MCV (fL)	85 (80.4–87.6)	84.65 (80.57–88.22)	0.52	
MCH (pg)	29.4 (27.9–30.4)	29.5 (27.32–30.6)	0.54	
HCT	41.61 ± 4.20	40.86 ± 4.19	0.07	
MCHC (g/dL)	34.6 (33.9–35)	34.45 (33.8–35.1)	0.57	
HDL (mg/dL)	39.39 ± 8.82	43.83 ± 10.13	<0.0001*	
LDL (mg/dL)	96.78 ± 25.39	100.76 ± 26.67	0.09	
ALP (U/L)	199 (160–252)	195 (159–231)	0.20	
ALT (U/L)	23 (16–34)	18 (13.97–27)	<0.0001*	
AST (U/L)	21 (18–27)	20 (17–25)	0.17	
HbA1c (mmol/mol)	5 (4.6–5.7)	4.4 (4–5)	<0.0001*	
SBP (mmHg)	125 (110–140)	115 (100–125)	<0.0001*	
DBP (mmHg)	80 (70–90)	70 (60–80)	<0.0001*	
MAP (mmHg)	96.27 ± 14.67	86.54 ± 13.31	<0.0001*	
BMI (kg/m^2^)	29.41 (26.34–33.11)	26.67 (23.49–30.30)	<0.0001*	
WHR	0.904 ± 0.066	0.874 ± 0.067	<0.0001*	
WHtR	0.568 ± 0.066	0.531 ± 0.070	<0.0001*	
Smoking				
Yes	301	165	1	
No	57	31		
Serum insulin levels (pmol/L)	10.93 (7.30–15.03)	9.04 (6.26–13.59)	0.008*	
HOMA index	2.93 (1.89–4.29)	2.13 (1.35–3.21)	<0.0001*	

*Note*: Chi‐square test (*p*‐value) was used for categorical variables (e.g., sex and diabetes), while *t*‐test (*p*‐value) was utilized for continuous variables (e.g., Age and FBS). BH denotes the corrected *p*‐value for multiple comparisons (Benjamini & Hochberg). Significance level was set at *p*‐value < 0.05 (*).

Abbreviations: ALP, Alkaline phosphatase; ALT, alanine transaminase; AST, aspartate aminotransferase; BMI, body mass index; CRP, c‐reactive protein; DBP, diastolic blood pressure; FBS, fast blood sugar; GGT, gamma glutamyl transferase; Hb, hemoglobin; HCT, Hematocrit; HDL, high‐density lipoprotein; HOMA, homeostatic model assessment; LDL, low‐density lipoprotein; MAP, mean arterial pressure; MCH, mean corpuscular hemoglobin; MCHC, mean cell hemoglobin concentration; MCV, mean corpuscular volume; SBP, Systolic blood pressure; TG, triglycerides; WHR, waist–hip ratio; WHtR, Waist‐To‐Height Ratio.

### 3.2 Relationships between demographic and clinical factors and IKZF3, lncRNA CDKN2B‐AS1, and microRNA‐LET7‐a‐2 genotypes

Analyzing HWE for *IKZF3* rs907091, microRNA*‐LET7* rs1143770, and lncRNA *CDKN2B‐AS1* rs1333045 in the control group showed that they were all in HWE. Besides, the results have been adjusted for confounders including sex, age, height, CRP, Hb, ALT, AST, ALP, GGT, MCV, MCH, HCT, MCHC, and smoking status. The genotype distribution and allele frequencies of *IKZF3*, microRNA*‐LET7‐a‐2*, and lncRNA *CDKN2B‐AS1* and also their genetic models between MetS patients and healthy controls are presented in Table 3. There were statistically significant associations of genotype and allelic variations for rs1333045 T>C (OR = 0.56, 95% CI 0.38–0.81, *p* = 0.002; OR = 0.71, 95% CI 0.55–0.92, *p* = 0.008), while rs907091 C>T and rs1143770 C>T did not show statistically significant associations between studied groups. In addition, results revealed that lncRNA *CDKN2B‐AS1* rs1333045T>C was significantly associated with a decreased risk of MetS progression under co‐dominant, dominant, and log‐additive models after adjustment (CT vs. TT: OR = 0.57, 95% CI 0.37–0.88, *p* = 0.007; CC vs. TT: OR = 0.47, 95% CI 0.26–0.84, *p* = 0.007, co‐dominant model, CT + CC vs. TT: OR = 0.66, 95% CI 0.39–1.10, *p* = 0.003, dominant model, and TT vs. TC vs. CC: OR = 0.66, 95% CI 0.50–0.88, *p* = 0.004, log‐additive model). Also, the frequency of the C allele in the patients' group was significantly higher than that in the healthy group (OR = 0.71, 95% CI 0.55–0.92, *p* = 0.008).

**TABLE 3:** Genotype and allele distributions of three variants and their association with the risk of metabolic syndrome under different genetic models.

Variant	Genotype	Study population		*p*‐Value^a^ (BH)	Genetic model		Adjusted OR (95% CI)	*p*‐Value^b^ (BH)
		Case	Control	Unadjusted OR (95% CI)				
*IKZF3*	TT	114 (32%)	51 (27.1%)	0.235 (0.352) 1.27 (0.86–1.87)	Co‐dominant	TC	1.19 (0.75–1.88)	0.486
	TC	173 (48.6%)	92 (48.9%)			CC	1.33 (0.76–2.30)	0.203
	CC	69 (19.4%)	45 (23.9%)		Dominant	TC + CC	1.23 (0.80–1.90)	0.346
	T	401 (56.3%)	194 (51.6%)	0.137 (0.274) 1.21 (0.94,1.55)	Recessive	CC	1.19 (0.75–1.89)	0.468
	C	311 (43.7%)	182 (48.4)		Overdominant	TC	1.05 (0.71–1.55)	0.80
					Log‐Additive	TT, TC, CC	1.15 (0.88–1.52)	0.30
*microRNA‐let7*	CC	131 (36.8%)	67 (35.6%)	0.789 (0.789) 1.05 (0.73–1.52)	Co‐dominant	CT	1.27 (0.82–1.96)	0.286
	CT	154 (43.3%)	92 (48.9%)			TT	0.80 (0.45–1.41)	0.481
	TT	71 (19.9%)	29 (15.4%)		Dominant	CT + TT	1.11 (0.74–1.67)	0.621
	C	416 (58.5%)	226 (60.1%)	0.592 (0.710) 0.93 (0.72,1.20)	Recessive	TT	0.70 (0.42–1.18)	0.171
	T	296 (41.5%)	150 (39.9%)		Overdominant	CT	1.36 (0.92–2.2)	0.12
					log‐Additive	CC, CT, TT	0.95 (0.72–1.24)	0.68
*CDKN2B*	TT	96 (27%)	75 (39.9%)	0.002* (0.012) 0.56 (0.38–0.81)	Co‐dominant	CT	0.57 (0.37–0.88)	0.007*
	TC	190 (53.4%)	83 (44.1%)			CC	0.47 (0.26–0.84)	0.007*
	CC	70 (19.7%)	30 (16%)		Dominant	CT + CC	0.66 (0.39–1.10)	0.003*
	T	382 (53.6%)	233 (62%)	0.008* (0.024) 0.71 (0.55–0.92)	Recessive	CC	0.66 (0.39–1.10)	0.105
	C	330 (46.4%)	143 (38%)		Overdominant	TC	0.75 (0.50–1.10)	0.13
					log‐Additive	TT, TC, CC	0.66 (0.50–0.88)	0.004*

*Note*: The statistical analysis for IKZF3 rs907091 and CDKN2B rs1333045 included Codominant (TT, TC, and CC), Dominant (TT vs. TC‐CC), Recessive (TT‐TC vs. CC), and Overdominant (TT‐CC vs. TC) models. For microRNA‐let7 rs1143770, the analysis encompassed Codominant (CC, CT, and TT), Dominant (CC vs. CT‐TT), Recessive (CC‐CT vs. TT), and Overdominant (TT‐CC vs. TC) models. *p*‐value^a^ was calculated using a two‐sided χ^2^ test for genotype distributions or allele frequencies, while *p*‐value^b^ was determined using logistic regression with different models. BH denotes the corrected *p*‐value for multiple comparisons (Benjamini & Hochberg), presented in parentheses. The significance level was set at *p*‐value <0.05 (*).

Besides, the association of SNPs with TG, cholesterol, CRP, HDL, HOMA, and HbA1c values was measured with adjustment of sex, age, height, CRP, Hb, ALT, AST, ALP, GGT, MCV, MCH, HCT, MCHC, and smoking status (Table 4). As shown in Table 4, there was a significant association between *IKZF3* rs907091 and TG, cholesterol, and HOMA in the patients' group and CRP in the healthy group (*p* = 0.013, 0.047, 0.011, and 0.008, respectively). In addition, a statistically significant association was observed between microRNA‐*LET7‐a‐2* and TG, cholesterol, CRP and HOMA (*p* = 0.032, 0.005, 0.022, and 0.006, respectively) in patients as well as in controls except for HOMA index (*p* = 0.039, 0.043, and 0.033, respectively). Although lncRNA *CDKN2B‐AS1* rs1333045 showed no significant association with any of the clinical parameters, in the combined model (patients and controls altogether), there were significant associations with HDL and HOMA index in the control group (*p* = 0.042 and 0.030, respectively).

**TABLE 4:** Association between variants and TG, Cholesterol, CRP, HDL, HOMA, and HbA1c values in all participants.

Factor	Cases			Controls			Combined		
	IKZF3	MicroRNA‐let7	CDKN2B	IKZF3	MicroRNA‐let7	CDKN2B	IKZF3	MicroRNA‐let7	CDKN2B
TG (mg/dL)	0.013*	0.032*	0.344	0.797	0.039*	0.835	0.020*	0.093	0.849
Cholesterol (mg/dL)	0.047*	0.005*	0.107	0.446	0.043*	0.249	0.038*	0.0008*	0.850
CRP (mg/dL)	0.788	0.022*	0.073	0.008*	0.033*	0.561	0.093	0.536	0.135
HDL (mg/dL)	0.076	0.469	0.196	0.254	0.880	0.367	0.048*	0.770	0.042*
HOMA	0.011*	0.943	0.496	0.857	0.556	0.924	0.054	0.733	0.911
HbA_1c_ (mmol/mol)	0.200	0.006*	0.571	0.337	0.096	0.191	0.958	0.039*	0.030*

*Note*: The statistical analysis involved determining whether the difference between means is statistically significant, rather than computing an OR. Significance level was set at *p*‐value <0.05 (*).

Abbreviations: CRP, c‐reactive protein; HDL, high‐density lipoprotein; HOMA, homeostatic model assessment; TG, triglycerides.

The associations between *IKZF3* rs907091 T>C, microRNA*‐LET7A2* rs1143770C>T, and lncRNA*‐CDKN2B‐AS1* rs1333045T>C genotypes and clinical parameters of MetS were investigated (Tables S1–S3). The results showed that the TT genotype at rs907091 T>C was significantly associated with an increase in TG, cholesterol, HOMA index, and insulin levels in the patients' group in comparison with those presenting with CC and CT genotypes. It also illustrated that TT genotype at rs907091 T>C was strongly associated with higher levels of LDL, CRP, and HTC in the control group compared to CC and CT genotypes. Furthermore, the results revealed that HbA1c and BMI in patients and CRP in the control group were higher in subjects having CC and CT genotypes for rs1143770C>T in comparison with those with the TT genotype. There were no statistically significant associations of rs1333045T>C with clinical criteria, except for HDL, which was higher in subjects with CC genotype in comparison with those with TT and CT genotypes in the control group.

### 3.3 In silico analysis of impacts of SNPs

The secondary structures of *IKZF3* and lncRNA *CDKN2B‐AS1* caused by rs907091 C>T and rs1333045T>C, respectively, were analyzed using the Sfold program and Srna tool, and results are presented in Table S4. The secondary structures, clustering, the free energy of hybridization (ΔGs), and multidimensional scaling (MDS) plots for both SNPs were calculated. Moreover, there were two clusters for each T and C alleles of *IKZF3* rs907091 C>T, while there were two and three clusters for T and C alleles of lncRNA *CDKN2B‐AS1* rs1333045T>C, respectively. Although rs1333045T>C did not show a significant difference in ΔGs for T (−62.70) and C (−65.80) alleles, *IKZF3* rs907091 C>T demonstrated a significant change in ΔGs between wild (T: −64.30) and mutant (C: −35.14) alleles. Tables 5 and 6 show the results of the prediction of microRNAs binding profile at *IKZF3* 3′UTR for both rs907091 alleles using miRSNP and miRSNPdb. Moreover, the results of RNAhybrid software for *IKZF3* rs907091 are illustrated in Table S5. There was a consensus normal binding site for *hsa‐let‐7a‐2‐3p*, which showed a decrease in minimum free energy (MFE) of the microRNA/mRNA duplex in the presence of the T allele which may facilitate *hsa‐let‐7a‐2‐3p* binding to *IKZF3* 3′UTR. Importantly, RNAhybrid revealed that *hsa‐miR‐4497* has a significantly lower MFE for C allele (−21.1) in comparison with the T allele (−15.8), resulting in stronger microRNA/mRNA duplex binding in the presence of the C allele. Correspondingly, miRSNP and miRSNPdb predicted consensus binding site losses for the T allele of rs907091. Besides, the effects of lncRNA *CDKN2B‐AS1* rs1333045T>C on the microRNA profile were predicted by the LncRNASNP2 program. As Table 7 shows, rs1333045T>C causes a gain of the binding site in lncRNA *CDKN2B‐AS1* for seven microRNAs.

**TABLE 5:** Predicting of microRNAs profile binding to *IKZF3* 3′UTR for alleles of rs907091 T>C by miRSNPs.

microRNAs	Effect	Allele	Score	Energy	Conservation
*hsa‐miR‐1266*	Break	T	140.00	−15.29	0.002
		A	NA	NA	NA
*hsa‐miR‐1266*	Break	T	140.00	−15.29	0.002
		C	NA	NA	NA
*hsa‐miR‐1266*	Break	T	140.00	−15.29	0.002
		G	NA	NA	NA
*hsa‐miR‐211‐3p*	Break	T	143.00	−16.54	0.001
		A	NA	NA	NA
*hsa‐miR‐211‐3p*	Break	T	143.00	−16.54	0.001
		C	NA	NA	NA
*hsa‐miR‐211‐3p*	Break	T	143.00	−16.54	0.001
		G	NA	NA	NA
*hsa‐miR‐3144‐5p*	Break	C	145.00	−17.46	0.001
		A	NA	NA	NA
*hsa‐miR‐3144‐5p*	Create	G	NA	NA	NA
		C	145.00	−17.46	0.001
*hsa‐miR‐3144‐5p*	Create	T	NA	NA	NA
		C	145.00	−17.46	0.001
*hsa‐miR‐3191‐5p*	Create	C	NA	NA	NA
		A	147.00	−20.51	0.002
*hsa‐miR‐3191‐5p*	Create	G	NA	NA	NA
		A	147.00	−20.51	0.002
*hsa‐miR‐3191‐5p*	Create	T	NA	NA	NA
		A	147.00	−20.51	0.002
*hsa‐miR‐326*	Create	C	NA	NA	NA
		A	153.00	−23.56	0.002
*hsa‐miR‐326*	Create	G	NA	NA	NA
		A	153.00	−23.56	0.002
*hsa‐miR‐326*	Create	T	NA	NA	NA
		A	153.00	−23.56	0.002
*hsa‐miR‐330‐5p*	Create	C	NA	NA	NA
		A	150.00	−21.21	0.002
*hsa‐miR‐330‐5p*	Create	G	NA	NA	NA
		A	150.00	−21.21	0.002
*hsa‐miR‐330‐5p*	Create	T	NA	NA	NA
		A	150.00	−21.21	0.002
*hsa‐miR‐4314*	Create	C	NA	NA	NA
		A	140.00	−15.81	0.000
*hsa‐miR‐4314*	Create	G	NA	NA	NA
		A	140.00	−15.81	0.000
*hsa‐miR‐4314*	Create	T	NA	NA	NA
		A	140.00	−15.81	0.000
*hsa‐miR‐4497*	Break	G	153.00	−25.87	0.000
		A	NA	NA	NA
*hsa‐miR‐4497*	Break	G	153.00	−25.87	0.000
		C	NA	NA	NA
*hsa‐miR‐4497*	Create	T	NA	NA	NA
		G	153.00	−25.87	0.000
*hsa‐miR‐4518*	Break	T	162.00	−23.66	0.002
		A	NA	NA	NA
*hsa‐miR‐4518*	Break	T	NA	NA	NA
		C	162.00	−23.66	0.002
*hsa‐miR‐4518*	Break	T	NA	NA	NA
		G	162.00	−23.66	0.002

*Note*: Profile of nine different microRNAs was found to be influenced by rs907091 T>C.

Abbreviation: NA, not analyzed.

**TABLE 6:** Predicting of microRNA profile binding to *IKZF3* 3′UTR for alleles of rs907091 T>C by miRSNPdb.

	Mature microRNA ID	MicroRNA Normal/Snp	Target gene ID	Target gene name	UTR type	Target SNP	RS ID
Consensus normal	*hsa‐let‐7a‐2‐3p*		ENSG00000161405	*IKZF3*	3′ UTR	301 C/T	rs907091
Consensus gains	*hsa‐miR‐3191‐5p*	Wild Type	ENSG00000161405	*IKZF3*	3′ UTR	301 C/T	rs907091
	*hsa‐miR‐326*	Wild Type	ENSG00000161405	*IKZF3*	3′ UTR	301 C/T	rs907091
	*hsa‐miR‐330‐5p*	Wild Type	ENSG00000161405	*IKZF3*	3′ UTR	301 C/T	rs907091
	*hsa‐miR‐4314*	Wild Type	ENSG00000161405	*IKZF3*	3′ UTR	301 C/T	rs907091
Consensus losses	*hsa‐miR‐4497*	Wild Type	ENSG00000161405	*IKZF3*	3′ UTR	301 C/T	rs907091

**TABLE 7:** Predicting of the microRNA profile for lncRNA *CDKN2B‐AS1* in the presence of rs1333045 T>C alleles through lncRNASNP2 tool.

MicroRNA ID	Energy (kCal/mol)	Binding start (target scan)	Binding end (target scan)	Binding start (miRanda)	Binding end (miRanda)	miRNA‐lncRNA interaction
*hsa‐miR‐5582‐5p*	−13.71	22,119,192	22,119,198	22,119,178	22,119,199	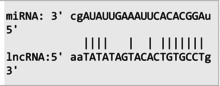
*hsa‐miR‐4755‐3p*	−15.22	22,119,195	22,119,202	22,119,181	22,119,202	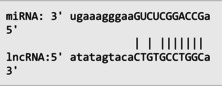
*hsa‐miR‐5006‐5p*	−21.18	22,119,196	22,119,202	22,119,182	22,119,203	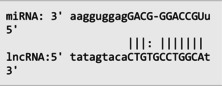
*hsa‐miR‐6715b‐5p*	−16.540001	22,119,193	22,119,199	22,119,181	22,119,200	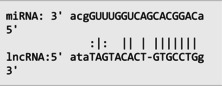
*hsa‐miR‐4673*	−17.16	22,119,194	22,119,200	22,119,182	22,119,201	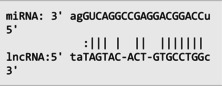
*hsa‐miR‐4645‐5p*	−16.110001	22,119,194	22,119,200	22,119,183	22,119,201	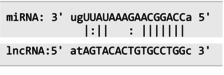
*hsa‐miR‐4269*	−19.780001	22,119,193	22,119,199	22,119,180	22,119,200	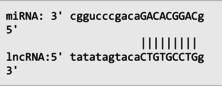

## 4 DISCUSSION

MetS is a multifactorial disorder which may be influenced by genetic polymorphisms involved in the regulation of the obesity process, blood pressure, blood sugar levels, and lipid metabolism,^30^, ^31^ as evidenced by studies on protein‐coding genes nitric oxide synthase (*NOS4*) cluster of differentiation 36 (*CD36*), BUD13 Homolog (*BUD13*), ZPR1 Zinc Finger (*ZPR1*), and apolipoprotein A5 (*APOA5*) SNPs in the Iranian population. However, in the present study, our focus shifted towards non‐coding RNA SNPs.^13^, ^14^, ^15^ Specifically, we investigated the association of *IKZF3* rs907091 C>T, microRNA‐*LET7‐a‐2* rs1143770C>T, and lncRNA *CDKN2B‐AS1* rs1333045T>C with susceptibility to MetS. The locus of the *CDKN2A/B* on chromosome 9p21 has been widely investigated and showed an association with a higher incidence of coronary artery calcium, ischemic stroke, obesity, T2DM, cardiomyopathy, myocardial infarction, coronary heart disease, and low HDL–cholesterol levels.^32^, ^33^, ^34^, ^35^, ^36^, ^37^ The results of the present study disclosed a significant difference in the allele and genotype distribution of rs1333045 between studied groups and also an association of the CC genotype with increased HDL levels compared to the TC + TT genotype (*p* = 0.038). Accordingly, a significant increase in the CC genotype has been reported in atrophic gastritis patients in comparison with controls (33.6% and 21.9%, respectively, *p* = 0.014), while similar results were not found for the gastric cancer group.^38^ Noticeably, other studies have shown no significant difference for rs1333045 alleles and genotypes between studied groups in autism spectrum disorders and multiple sclerosis, highlighting the probable role of sample size and ethnicity for this discrepancy.^39^, ^40^ Furthermore, a meta‐analysis on 19 SNPs in five common lncRNAs, including *CDKN2B‐AS1*, showed no significant association of rs1333045 with cancer risk. However, rs1333048 and rs4977574 were significantly associated with the risk of overall cancer in all genetic models.^41^

Our findings also illustrated the association of lncRNA *CDKN2B‐AS1* rs1333045T>C under the codominant, dominant, and log‐additive models with decreased risk of MetS after *P* value correction by the Benjamini & Hochberg method. In this regard, Hul et al.^42^ showed a significant difference between allele frequency in coronary heart disease patients and the healthy group and elevated levels of LDL in the CC genotype compared to the TC+TT genotype (*p* = 0.032). Noteworthy, HDL has been long known as an anti‐thrombotic factor reducing cardiovascular disease risk because of its key role in cholesterol transportation from peripheral tissues into the liver under a process known as the reverse cholesterol transport (RCT) system.^43^, ^44^, ^45^ Herein, according to our findings, it is not surprising that subjects carrying CC with higher levels of HDL are at lower risk for MetS development. A study that genotyped 8 *CDKN2B* SNPs in 4650 Saudi Arabs to assess their association with cardiovascular risk found that the T allele of rs1333045 [0.54(0.48–0.61); *p* < 0.0001] was significantly associated with coronary artery disease (CAD) after adjusting for other risk factors. This finding suggests that the *CDKN2B* gene locus plays a critical role in cardiovascular risk among Arabs.^46^ Consistently, it has been reported that the T allele at rs1333045 is statistically associated with increased susceptibility to coronary artery disease (*p* < 0.0001), T2DM (*p* = 0.048), and myocardial infarction (*p* = 1.15E−08).^47^, ^48^, ^49^ Noteworthy in silico analysis showed that lncRNA *CDKN2B* rs1333045T>C causes to gain microRNA target sites for seven microRNAs and probably has the strongest and weakest binding to *hsa‐miR‐5006‐5p* and *hsa‐miR‐5582‐5p*, respectively. Interestingly, Hyun‐Ju An et al. discovered *hsa‐miR‐5582‐5p* as a novel microRNA that acts as a tumor suppressor. They found that *hsa‐miR‐5582‐5p* can induce apoptosis and cell cycle arrest in cancer cells, but not in normal cells. The researchers identified three target proteins, GRB2‐associated binding protein 1 (GAB1), SRC homology 2 domain containing 1 (SHC1), and growth factor receptor‐bound protein 2 (CDK2), which are directly regulated by *hsa‐miR‐5582‐5p*. Knocking down the corresponding genes mimicked the effects of *miR‐5582‐5p* on apoptosis and cell cycle arrest. They showed that the expression of *hsa‐miR‐5582‐5p* was lower in tumor tissues compared to adjacent normal tissues in colorectal cancer patients, while the expression of the target proteins showed opposite patterns. Injecting a mimic of *hsa‐miR‐5582‐5p* or inducing its expression in tumor cells suppressed tumor growth in xenografts. These findings suggest that *hsa‐miR‐5582‐5p* has a novel tumor suppressive function and could potentially be used for tumor control.^50^

IKZF3 as a hematopoietic‐specific transcription factor plays a fundamental role in the regulation of lymphocyte development, especially B lymphocyte proliferation and differentiation.^16^ Moreover, genome‐wide association studies (GWASs) have shown that *IKZF3* is associated with lipid metabolism disorders.^17^ The first study of the impact of *IKZF3* rs907091 on disease development was conducted by Verlaan et al. and revealed the significant association of the T allele at *IKZF3* rs907091 with susceptibility to autoimmune disease.^49^ Our findings revealed no significant difference in rs907091 alleles and genotypes between the studied groups. On the contrary, a significant lower frequency in the CC genotype and C allele has been reported in the systemic lupus erythematous group in comparison with the control group (*p* = 0.001). Besides, the frequency of the T allele was reported to be significantly higher in patients compared to the control group (*p* = 0.015) and probably increased risk of systematic lupus erythematous progression.^51^ Nevertheless, our results showed that the TT genotype at *IKZF3* rs907091 is associated with increased levels of TG, cholesterol, insulin, and HOMA and also new binding site creation for *hsa‐miR‐326* in the presence of the T allele. Consistently, Rechardson et al.^52^ demonstrated that the T allele reduces the expression levels of *IKZF3* in T‐cells by creating a microRNA seed region for *hsa‐miR‐326*.^52^ Moreover, in silico analyses revealed that rs907091 T strongly changed the secondary structure of *IKZF3*, and the ΔG hybridization difference in the presence of C and T alleles (−35.14 and −64.30, respectively) was remarkable. *hsa‐miR‐1266* also had the strongest binding to *IKZF3* (ΔGs = −37.5 for the T allele and ΔGs = −36.8 for C allele), while the highest difference for ΔGs was related to *hsa‐miR‐LET7‐a‐2* (T allele: ΔGs = −16.6) and (C allele: ΔGs = −20.1) alleles. Strikingly, *hsa‐miR‐1266* has been shown to be associated with proliferation, migration, and invasion through targeting DAB2IP in HeLa and SiHa cells. It was also shown to enhance cervical cancer tumorigenesis and metastasis in the cervical cancer nude mice model.^53^

MicroRNA*‐LET7‐a2* has a key role in different disorders including T2DM, diabetic neuropathy, inflammation, and cerebral and cardiovascular conditions.^54^, ^55^ There was no significant difference in the allele and genotype frequency of rs1143770C>T between the studied groups. Inconsistently, Heidari et al. revealed a statistically significant higher frequency of CT and TT genotypes in patients with papillary thyroid carcinoma compared to controls, and these genotypes were associated with a 1.9‐fold and 2.2‐fold higher risk of disease, respectively.^56^ The results of the present study showed the association between CC and CT genotypes of *LET7‐a2* rs1143770C>T with increased levels of HbA1C, BMI, and CRP and probably higher risk of MetS susceptibility. Consistent with our results, the rs1143770 TT genotype was reported to be associated with significantly better overall survival and disease‐free survival in patients with surgically treated non‐small cell lung cancer (*p* = 0.01).^57^ In addition, investigating the association between *LET7a‐2* rs1143770 and papillary thyroid carcinoma (PTC) susceptibility demonstrated that CT and TT genotypes were associated with a higher risk of PTC, as well as a higher risk of N1 stage in PTC patients.^56^ On the other hand, Wang et al. revealed that subjects with the rs1143770 CC genotype were at reduced risk of ischemic stroke in comparison with individuals with the TT genotype.^58^ In addition, Zhou et al. reported a significant increase of CT + TT genotype frequencies in diabetic nephropathy and diabetes mellitus in comparison with the control group (*p* = 0.007 and 0.040, respectively) and a significant association with increased risk for diabetic nephropathy.^55^ These controversial results may be due to different sample sizes, methods, and population origins among mentioned studies and should be validated with a larger sample size and similar conditions.

The present study disclosed a significant association between the TT genotype at *IKZF3* rs907091 T>C and the CC genotype at microRNA*‐LET7‐a2* rs1143770 C>T with increased risk of Mets, while the CC genotype for rs1333045 T>C was associated with decreased risk of Mets. In this context, subjects with the TT genotype for rs907091 T>C showed higher levels of TG, cholesterol, serum insulin levels, and HOMA index in comparison with those with TC and CC genotypes. Notably, the T allele at rs907091 T>C created a new binding site for hsa‐miR‐326 and also caused much strong binding to hsa‐miR‐LET7‐a‐2. Moreover, HbA1C and BMI were disclosed to be higher in patients with the CC and CT genotypes for rs1143770 C>T compared to patients with the TT genotype contributing to an increased risk of MetS development. In addition, the CC genotype for rs1333045 T>C was clearly associated with increased HDL levels in subjects who had CC genotype in comparison with subjects with TT and CT genotypes and probably decreased MetS susceptibility risk. It is worth mentioning that lncRNA*‐CDKN2B‐AS1* rs1333045 T>C created new binding sites for seven microRNAs of which hsa‐miR‐5006‐5p and hsa‐miR‐5582‐5p had the strongest and weakest binding potential, respectively. Notably, after SNP interaction analyses to assess whether there were synergistic or modifying effects between these gene, we did not observe any statistically significant interactions between the studied genes. Although the absence of significant interactions suggests that the effects of these genetic variants may act independently or have minimal interaction in our studied population, it is important to note that non‐significant findings do not necessarily indicate the absence of interactions in a broader context or in different populations. Interactions between genes can be complex and influenced by various factors, including genetic backgrounds and environmental exposures.

It is worth noting that the strengths of the present study lie in investigating the association of specific genetic polymorphisms in *IKZF3*, *LET7‐a2*, and *CDKN2B‐AS1*, with MetS pathogenesis under different genetic models in a large sample size for the first time. Furthermore, genotype–phenotype was assessed to investigate the association of specific genetic polymorphisms with various biochemical, hematological, clinical, and physiological parameters. We also utilized different bioinformatics databases to analyze the impact of these polymorphisms on mRNA and microRNA profiles, providing a broad overview on the crucial role of ncRNAs in MetS pathogenesis. On the other hand, one of the important disadvantages of the present study is its reliance on a single population (Mazandarn province) for analysis, which may limit the generalizability of the findings. Additionally, the study does not use methods to analyze the possible impact of these polymorphisms on the expression levels of either IKZF3, LET7‐a2, and CDKN2B‐AS1 genes or proteins. The lack of in vivo analyses for the data recruited from bioinformatics tools is another weakness of this study. Taken together, identifying genetic polymorphisms which put subjects at higher risk of MetS may pave the road to approving the efficient predictive biomarkers at the early stage of MetS development. Further evaluation of these polymorphisms in other populations could be helpful in this regard.

## AUTHOR CONTRIBUTIONS

Haleh Akhavan‐Niaki and Alireza Paniri contributed to the design and conception of the present study. Patient physical examination was conducted by Farhad Zamani and Mansooreh Maadi. Farhad Zamani, Mansooreh Maadi, Nima Motamed, and Mohsen Asouri contributed to data collection and cohort processing. Mohammad Mahdi Hosseini perfomed statistical analyses of results, tables designing, and worked on the manuscript. Alireza Paniri, Sadegh Fattahi, and Galia Amirbozorgi performed exprimental investigations. Alireza Paniri performed bioinformatic analyses. Alireza Paniri and Mohammad Mahdi Hosseini contributed to manuscript drafting preparation. Haleh Akhavan‐Niaki supervised the present study.

## FUNDING INFORMATION

This research received grant from Babol University of Medical Sciences.

## CONFLICT OF INTEREST STATEMENT

Authors declare no conflict of interest.

## Supporting information


Table S1

## Data Availability

The data that support the findings of this study are available from the corresponding author upon reasonable request.
