# Supplementary material for: Genetic variations in IKZF3, LET7‐a2, and CDKN2B‐AS1: Exploring associations with metabolic syndrome susceptibility and clinical manifestations
Source: J Clin Lab Anal. 2024 Jan 9;38(1-2):e24999. doi: 10.1002/jcla.24999 (PMC10829692; doi:10.1002/jcla.24999)
Supplement: Supplementary file 1 — Table S1 [file JCLA-38-e24999-s001.docx]

**Table S1: Association between *IKZF3* rs907091 C>T and various clinical parameters**

| ***IKZF3* rs907091 C>T** | | | | | | | | |
| --- | --- | --- | --- | --- | --- | --- | --- | --- |
|  | **Case**s | | | | **Control**s | | | |
| **Parameters** | CC (69) | CT (173) | TT (114) | p-value | CC (45) | CT (92) | TT (51) | p-value |
| **Age** | 48.97±14.51 | 50.26±14.28 | 50.85±14.91 | 0.694 | 52.84±14.55 | 47.20±16.44 | 51.86±13.00 | 0.067 |
| **Waist circumference (Cm)** | 95.02±10.50 | 93.6±9.34 | 93.08±9.12 | 0.401 | 86.42±8.76 | 87.61±10.91 | 88.27±13.16 | 0.712 |
| **Height (Cm)** | 166.26±9.24 | 165.09±9.13 | 165.01±10.20 | 0.642 | 164.77±9.30 | 166.35±10.77 | 163.05±10.67 | 0.191 |
| **Weight (Kg)** | 84.66±15.58 | 81.55±14.90 | 81.22±14.24 | 0.262 | 72.90±13.61 | 75.96±16.05 | 73.08±17.59 | 0.445 |
| **Hip (Cm)** | 105.31±8.43 | 103.5665±8.92 | 103.0000±7.55 | 0.186 | 99.44±7.99 | 100.69±9.16 | 99.64±11.21 | 0.709 |
| **FBS (mg/dL)** | 124.07±34.93 | 131.73±53.60 | 126.65±48.01 | 0.475 | 105.31±33.32 | 101.00±33.21 | 105.03±30.33 | 0.68 |
| **TG (mg/dL)** | 149.17±100.75 | 155.05±84.78 | 185.97±146.74 | **0.034*** | 117.13±63.68 | 122.55±93.77 | 113.47±64.17 | 0.799 |
| **Cholesterol (mg/dL)** | 173.94±46.53 | 183.85±40.29 | 193.09±41.73 | **0.010*** | 185.06±36.63 | 180.24±45.42 | 190.79±42.96 | 0.38 |
| **HDL (mg/dL)** | 37.91±5.99 | 39.32±10.01 | 40.33±8.30 | 0.199 | 43.15±10.33 | 43.20±9.18 | 46.29±11.68 | 0.181 |
| **LDL (mg/dL)** | 94.56±25.57 | 98.05±26.06 | 97.51±25.02 | 0.626 | 102.57±27.16 | 96.38±25.08 | 110.39±27.41 | **0.01*** |
| **HbA_1_c (mmol/mol)** | 5.13±0.89 | 5.21±0.88 | 5.13±0.96 | 0.705 | 4.42±0.90 | 4.33±0.82 | 4.52±0.91 | 0.46 |
| **HOMA index** | 2.68±1.64 | 3.35±2.17 | 3.78±2.76 | **0.008*** | 2.74±3.23 | 2.62±1.73 | 2.46±2.50 | 0.85 |
| **ALP (U/L)** | 229.28±119.21 | 212.00±87.82 | 202.15±61.52 | 0.137 | 199.61±100.80 | 223.28±122.97 | 192.50±53.87 | 0.189 |
| **ALT (U/L)** | 27.56±20.44 | 28.53±19.57 | 27.46±19.33 | 0.887 | 19.57±10.53 | 23.94±15.06 | 22.30±16.74 | 0.264 |
| **AST (U/L)** | 24.34±13.32 | 23.90±10.35 | 24.46±14.32 | 0.927 | 21.18±7.65 | 23.91±12.43 | 22.40±9.99 | 0.368 |
| **GGT (U/L)** | 28.50±13.29 | 33.45±23.37 | 34.02±20.76 | 0.203 | 25.62±10.81 | 28.04±19.07 | 25.35±12.91 | 0.573 |
| **CRP (mg/dL)** | 2.07±2.58 | 2.78±3.16 | 2.66±3.04 | 0.26 | 2.03±2.80 | 1.56±1.91 | 2.98±3.40 | **0.010*** |
| **Hb (g/dL)** | 14.57±1.44 | 14.32±1.57 | 14.35±1.78 | 0.544 | 13.52±1.97 | 14.34±1.45 | 14.07±1.55 | **0.024*** |
| **MCV (fL)** | 82.28±7.90 | 82.55±8.50 | 82.87±7.78 | 0.891 | 81.33±10.08 | 82.99±7.94 | 84.36±8.39 | 0.36 |
| **MCH (pg)** | 28.27±3.26 | 28.38±3.41 | 28.58±3.15 | 0.807 | 27.88±3.93 | 28.60±3.20 | 29.03±3.50 | 0.271 |
| **HCT** | 42.26±3.50 | 41.74±3.99 | 41.53±4.44 | 0.502 | 39.64±5.08 | 41.57±3.71 | 40.76±3.96 | **0.040*** |
| **MCHC (g/dL)** | 34.45±1.00 | 34.31±1.02 | 34.43±1.00 | 0.51 | 33.99±1.01 | 34.34±1.85 | 34.32±1.12 | 0.406 |
| **Ins (pmol/L)** | 10.44±5.96 | 12.00±6.70 | 12.98±6.57 | **0.043*** | 10.60±10.23 | 10.78±6.21 | 9.42±6.30 | 0.567 |
| **SBP (mmHg)** | 129.50±18.75 | 128.17±22.35 | 127.46±21.37 | 0.822 | 114.84±17.05 | 117.53±18.46 | 112.72±16.21 | 0.279 |
| **DBP (mmHg)** | 79.92±11.92 | 80.78±13.42 | 80.78±13.07 | 0.887 | 69.66±12.58 | 73.47±13.70 | 70.78±11.50 | 0.214 |
| **MAP (mmHg)** | 96.45±13.23 | 96.57±15.11 | 96.34±14.70 | 0.991 | 84.72±12.50 | 88.16±14.34 | 84.76±11.80 | 0.213 |
| **BMI (Kg/m^2^)** | 30.62±5.02 | 29.97±5.19 | 29.90±4.93 | 0.609 | 26.87±4.63 | 27.04±5.09 | 27.45±6.13 | 0.83 |
| **WHR** | 0.90±0.06 | 0.90±0.06 | 0.90±0.07 | 0.949 | 0.86±0.06 | 0.86±0.06 | 0.88±0.07 | 0.372 |
| **WHtR** | 0.57±0.06 | 0.56±0.06 | 0.56±0.06 | 0.82 | 0.52±0.05 | 0.52±0.07 | 0.54±0.08 | 0.414 |

One-way ANOVA was performed to analyze the differences between groups for continuous variables (e.g., Age, FBS, BMI, ...). Significance level was set at p-value < 0.05 (*). **FBS**: fast blood sugar; **TG**: triglycerides; **GGT**: gamma glutamyl transferase; **CRP**:  c-reactive protein; **Hb**: hemoglobin; **MCV**: mean corpuscular volume; **MCH**: mean corpuscular hemoglobin; **HCT**: Hematocrit; **MCHC**: mean cell hemoglobin concentration; **HDL**: high-density lipoprotein; **LDL**: low-density lipoprotein; **ALP**: Alkaline phosphatase; **ALT**: alanine transaminase: **AST**: aspartate aminotransferase; **SBP**: Systolic blood pressure; **DBP**: diastolic blood pressure; **MAP**: mean arterial pressure ;**BMI**: body mass index; **WHR**: waist–hip ratio; **WHtR**: Waist- To- Height Ratio; **HOMA**: homeostatic model assessment.

**Table S2. Association between microRNA*-let7* rs1143770C>T and various clinical parameters.**

| **microRNA-*let7* rs1143770C>T** | | | | | | | | |
| --- | --- | --- | --- | --- | --- | --- | --- | --- |
|  | **Cases** | | | | **Controls** | | | |
| **Parameters** | CC (131) | CT (154) | TT (71) | p-value | CC (67) | CT (92) | TT (29) | p-value |
| **Age** | 50.32±14.62 | 50.09±14.07 | 50.22±15.40 | 0.991 | 52.58±15.08 | 48.47±15.82 | 47.68±13.37 | 0.177 |
| **Waist circumference (Cm)** | 94.56±10.86 | 93.42±8.91 | 92.81±.93 | 0.402 | 87.91±12.15 | 87.61±10.54 | 86.20±10.42 | 0.78 |
| **Height (Cm)** | 164.08±9.54 | 165.53±9.50 | 167.01±9.22 | 0.103 | 163.65±10.40 | 165.92±10.31 | 165.72±11.00 | 0.379 |
| **Weight (Kg)** | 83.18±17.37 | 81.32±13.60 | 81.53±12.15 | 0.544 | 72.98±16.61 | 75.40±16.42 | 74.82±12.69 | 0.637 |
| **Hip (Cm)** | 104.74±9.41 | 103.21±8.02 | 102.94±7.19 | 0.212 | 99.13±10.03 | 100.54±9.50 | 101.00±8.09 | 0.563 |
| **FBS (mg/dL)** | 134.14±51.17 | 123.09±43.44 | 130.42±53.84 | 0.152 | 103.88±33.73 | 104.93±34.70 | 95.65±10.39 | 0.395 |
| **TG (mg/dL)** | 156.35±83.60 | 157.91±100.64 | 190.38±165.73 | 0.081 | 133.41±102.80 | 111.25±66.55 | 108.93±47.92 | 0.172 |
| **Cholesterol (mg/dL)** | 178.96±42.75 | 189.22±42.13 | 186.81±38.54 | 0.114 | 179.71±43.31 | 186.80±42.47 | 186.13±43.13 | 0.583 |
| **HDL (mg/dL)** | 39.28±8.34 | 39.23±9.75 | 39.84±7.67 | 0.881 | 44.65±10.08 | 44.16±10.68 | 42.17±9.13 | 0.545 |
| **LDL (mg/dL)** | 96.83±25.19 | 95.16±26.33 | 102.30±24.36 | 0.148 | 100.38±27.41 | 103.58±26.19 | 98.51±27.38 | 0.6 |
| **HbA_1_c (mmol/mol)** | 5.33±0.85 | 5.09±0.90 | 5.03±0.99 | **0.031*** | 4.37±0.97 | 4.51±0.76 | 4.15±0.88 | 0.139 |
| **HOMA index** | 3.38±2.22 | 3.29±2.12 | 3.47±2.85 | 0.852 | 2.35±1.71 | 2.82±2.89 | 2.48±1.76 | 0.457 |
| **ALP (U/L)** | 208.89±78.09 | 214.04±86.83 | 214.40±106.95 | 0.866 | 202.15±65.61 | 216.43±121.17 | 202.17±113.11 | 0.649 |
| **ALT (U/L)** | 25.84±16.59 | 29.26±23.28 | 29.35±15.88 | 0.287 | 20.76±9.44 | 23.58±16.16 | 22.45±18.66 | 0.5 |
| **AST (U/L)** | 23.19±12.49 | 24.14±11.79 | 26.02±12.92 | 0.301 | 22.00±6.69 | 22.56±8.85 | 25.52±19.97 | 0.329 |
| **GGT (U/L)** | 32.82±24.45 | 33.90±21.25 | 30.11±11.43 | 0.492 | 27.06±13.30 | 25.99±12.33 | 28.36±27.51 | 0.784 |
| **CRP (mg/dL)** | 2.56±2.96 | 2.42±3.00 | 3.07±3.16 | 0.336 | 2.77±3.05 | 1.87±2.55 | 1.06±1.43 | **0.010*** |
| **Hb (g/dL)** | 14.41±1.60 | 14.25±1.67 | 14.59±1.51 | 0.329 | 14.00±1.72 | 14.09±1.71 | 14.15±1.23 | 0.907 |
| **MCV (fL)** | 82.80±8.03 | 82.16±8.80 | 83.16±6.87 | 0.658 | 84.52±9.26 | 82.03±8.12 | 83.19±8.57 | 0.21 |
| **MCH (pg)** | 28.50±3.19 | 28.21±3.57 | 28.72±2.84 | 0.538 | 28.94±3.77 | 28.29±3.29 | 28.45±3.43 | 0.514 |
| **HCT** | 41.84±4.03 | 41.45±4.14 | 42.31±3.88 | 0.341 | 40.89±4.30 | 40.75±4.44 | 41.26±3.19 | 0.851 |
| **MCHC (g/dL)** | 34.35±0.97 | 34.35±1.09 | 34.48±0.91 | 0.642 | 34.15±1.14 | 34.31±1.85 | 34.27±0.88 | 0.794 |
| **Ins (pmol/L)** | 12.09±6.81 | 11.82±6.43 | 12.26±6.45 | 0.889 | 9.76±6.18 | 10.73±8.28 | 10.58±7.05 | 0.717 |
| **SBP (mmHg)** | 129.96±23.30 | 128.00±20.47 | 125.39±19.25 | 0.344 | 118.32±16.20 | 114.80±18.56 | 111.72±16.97 | 0.201 |
| **DBP (mmHg)** | 80.72±13.86 | 80.87±11.99 | 79.85±13.62 | 0.856 | 73.35±12.22 | 72.11±12.27 | 67.41±15.67 | 0.112 |
| **MAP (mmHg)** | 97.13±15.76 | 96.58±13.66 | 95.03±14.42 | 0.617 | 88.34±12.42 | 86.34±13.11 | 882.18±15.24 | 0.114 |
| **BMI (Kg/m^2^)** | 30.91±5.78 | 29.74±4.2 | 29.26±3.911 | **0.047*** | 27.22±5.70 | 27.33±5.27 | 27.30±4.32 | 0.99 |
| **WHR** | 0.90±0.06 | 0.90±0.06 | 0.90±0.06 | 0.886 | 0.88±0.07 | 0.87±0.06 | 0.85±0.06 | 0.07 |
| **WHtR** | 0.57±0.07 | 0.56±0.06 | 0.55±0.05 | 0.091 | 0.53±0.07 | 0.52±0.06 | 0.52±0.06 | 0.518 |

One-way ANOVA was performed to analyze the differences between groups for continuous variables (e.g., Age, FBS, BMI, ...). Significance level was set at p-value < 0.05 (*). **FBS**: fast blood sugar; **TG**: triglycerides; **GGT**: gamma glutamyl transferase; **CRP**:  c-reactive protein; **Hb**: hemoglobin; **MCV**: mean corpuscular volume; **MCH**: mean corpuscular hemoglobin; **HCT**: Hematocrit; **MCHC**: mean cell hemoglobin concentration; **HDL**: high-density lipoprotein; **LDL**: low-density lipoprotein; **ALP**: Alkaline phosphatase; **ALT**: alanine transaminase: **AST**: aspartate aminotransferase; **SBP**: Systolic blood pressure; **DBP**: diastolic blood pressure; **MAP**: mean arterial pressure ;**BMI**: body mass index; **WHR**: waist–hip ratio; **WHtR**: Waist- To- Height Ratio; **HOMA**: homeostatic model assessment.

**Table S3. Association between lncRNA *CDKN2B* rs1333045T>C and various clinical parameters.**

| **lncRNA *CDKN2B* rs1333045T>C** | | | | | | | | |
| --- | --- | --- | --- | --- | --- | --- | --- | --- |
|  | **Cases** | | | | **Controls** | | | |
| **Parameters** | CC (70) | CT (190) | TT (96) | p-value | CC (30) | CT (83) | TT (75) | p-value |
| **Age** | 49.02±13.4 | 49.62±14.90 | 52.21±14.40 | 0.271 | 45.96±13.21 | 50.63±15.19 | 50.45±16.06 | 0.322 |
| **Waist circumference (Cm)** | 93.34±9.94 | 93.05±9.47 | 95.31±9.15 | 0.155 | 85.16±11.21 | 87.74±10.53 | 88.18±11.62 | 0.439 |
| **Height (Cm)** | 166.00±9.04 | 165.15±9.86 | 165.06±9.15 | 0.786 | 166.26±8.3 | 164.87±10.05 | 164.84±11.65 | 0.798 |
| **Weight (Kg)** | 82.15±15.37 | 81.32±13.92 | 83.40±16.20 | 0.535 | 74.50±17.47 | 74.03±14.31 | 74.89±17.17 | 0.945 |
| **Hip (Cm)** | 103.31±8.88 | 103.63±7.97 | 104.19±9.01 | 0.784 | 100.33±11.23 | 99.86±8.38 | 100.29±9.97 | 0.952 |
| **FBS (mg/dL)** | 124.67±42.26 | 130.08±51.40 | 128.60±47.79 | 0.73 | 98.166±19.19 | 107.57±40.41 | 100.18±25.58 | 0.237 |
| **TG (mg/dL)** | 153.54±77.95 | 163.29±120.30 | 172.33±115.83 | 0.564 | 144.33±128.08 | 111.22±60.76 | 116±72.06 | 0.145 |
| **Cholesterol (mg/dL)** | 177.70±46.78 | 185.73±39.86 | 188.60±41.46 | 0.237 | 190.07±54.60 | 187.41±40.84 | 178.72±39.66 | 0.333 |
| **HDL (mg/dL)** | 38.70±4.14 | 39.23±7.95 | 40.13±11.32 | 0.56 | 47.10±11.19 | 42.04±9.64 | 45.00±10.14 | **0.038*** |
| **LDL (mg/dL)** | 95.88±27.16 | 95.95±24.69 | 100.63±26.15 | 0.308 | 101.30±25.79 | 102.60±30.60 | 100.77±22.52 | 0.91 |
| **HbA_1_c (mmol/mol)** | 5.24±0.87 | 5.12±0.98 | 5.21±0.96 | 0.592 | 4.33±0.91 | 4.52±0.91 | 4.30±0.78 | 0.266 |
| **HOMA index** | 3.41±2.06 | 3.28±2.37 | 3.47±2.41 | 0.802 | 2.48±.51 | 2.69±2.31 | 2.56±2.70 | 0.908 |
| **ALP (U/L)** | 213.63±61.29 | 214.43±102.37 | 206.78±73.98 | 0.783 | 192.21±68.66 | 213.22±120.02 | 211.20±95.20 | 0.639 |
| **ALT (U/L)** | 30.19±24.79 | 27.84±19.57 | 26.67±14.77 | 0.521 | 21.72±14.82 | 23.91±15.65 | 21.08±13.40 | 0.465 |
| **AST (U/L)** | 25.64±16.12 | 23.61±10.17 | 24.14±12.82 | 0.503 | 20.15±5.71 | 23.19±8.78 | 23.45±13.76 | 0.358 |
| **GGT (U/L)** | 35.85±27.04 | 32.22±20.48 | 31.24±16.06 | 0.371 | 24.86±13.11 | 28.63±19.64 | 25.39±11.62 | 0.38 |
| **CRP (mg/dL)** | 2.13±2.60 | 2.57±2.98 | 3.02±3.34 | 0.178 | 1.47±2.11 | 2.09±2.70 | 2.25±2.80 | 0.415 |
| **Hb (g/dL)** | 14.50±1.75 | 14.36±1.67 | 14.32±1.40 | 0.753 | 13.93±1.65 | 14.07±1.67 | 14.12±1.62 | 0.881 |
| **MCV (fL)** | 81.72±9.98 | 82.83±7.96 | 6.91±82.20 | 0.601 | 82.20±9.22 | 82.78±8.32 | 83.73±8.80 | 0.668 |
| **MCH (pg)** | 28.09±4.05 | 28.48±3.21 | 28.55±2.80 | 0.64 | 28.14±3.73 | 28.40±3.38 | 28.84±3.51 | 0.597 |
| **HCT** | 42.28±4.35 | 41.65±4.14 | 41.64±3.64 | 0.5 | 40.26±4.69 | 41.03±4.16 | 40.95±4.10 | 0.694 |
| **MCHC (g/dL)** | 34.26±1.20 | 34.39±0.98 | 34.42±0.91 | 0.586 | 34.32±1.02 | 34.09±1.95 | 34.39±1.02 | 0.452 |
| **Ins (pmol/L)** | 12.94±7.41 | 11.42±6.10 | 12.48±6.72 | 0.183 | 10.46±6.04 | 10.19±5.83 | 10.53±9.22 | 0.956 |
| **SBP (mmHg)** | 127.92±21.39 | 126.48±21.44 | 131.81±20.85 | 0.136 | 110.50±14.88 | 115.65±14.69 | 117.54±20.97 | 0.179 |
| **DBP (mmHg)** | 81.78±14.01 | 79.34±13.23 | 82.29±11.58 | 0.137 | 70.00±12.03 | 71.08±11.26 | 73.40±14.82 | 0.372 |
| **MAP (mmHg)** | 97.16±15.50 | 95.05±14.82 | 98.79±13.20 | 0.111 | 83.50±11.95 | 85.93±11.40 | 88.11±15.52 | 0.252 |
| **BMI (Kg/m^2^)** | 29.87±5.14 | 29.88±4.85 | 30.61±5.43 | 0.483 | 26.91±5.98 | 27.26±4.99 | 27.46±5.33 | 0.888 |
| **WHR** | 0.90±0.06 | 0.89±0.06 | 0.91±0.06 | 0.121 | 0.84±0.05 | 0.87±0.07 | 0.87±0.06 | 0.0789 |
| **WHtR** | 0.56±0.06 | 0.56±0.06 | 0.57±0.06 | 0.22 | 0.51±0.06 | 0.53±0.07 | 0.53±0.07 | 0.292 |

One-way ANOVA was performed to analyze the differences between groups for continuous variables (e.g., Age, FBS, BMI, ...). Significance level was set at p-value < 0.05 (*). **FBS**: fast blood sugar; **TG**: triglycerides; **GGT**: gamma glutamyl transferase; **CRP**:  c-reactive protein; **Hb**: hemoglobin; **MCV**: mean corpuscular volume; **MCH**: mean corpuscular hemoglobin; **HCT**: Hematocrit; **MCHC**: mean cell hemoglobin concentration; **HDL**: high-density lipoprotein; **LDL**: low-density lipoprotein; **ALP**: Alkaline phosphatase; **ALT**: alanine transaminase: **AST**: aspartate aminotransferase; **SBP**: Systolic blood pressure; **DBP**: diastolic blood pressure; **MAP**: mean arterial pressure ;**BMI**: body mass index; **WHR**: waist–hip ratio; **WHtR**: Waist- To- Height Ratio; **HOMA**: homeostatic model assessment.

**Table S4: The results of Srna tool of Sfold program for all SNPs. MDS plot, multi-dimensional scaling plot.** Two clusters were predicted for both alleles of *IKZF3* rs907091 C>T. Moreover, 2 and 3 clusters were found for T and C alleles of lncRNA *CDKN2B-AS1* rs1333045T>C, respectively. Although no significant difference in ΔGs was observed between T and C alleles of rs1333045T>C, *IKZF3* rs907091 C>T showed a significant difference in ΔGs between T and C alleles. MDS plot: multi-dimensional scaling plot.

| **SNP** | **Allele** | **Ensemble centroid structure diagram** | **MDS pl** |
| --- | --- | --- | --- |
| **rs907091** | T | 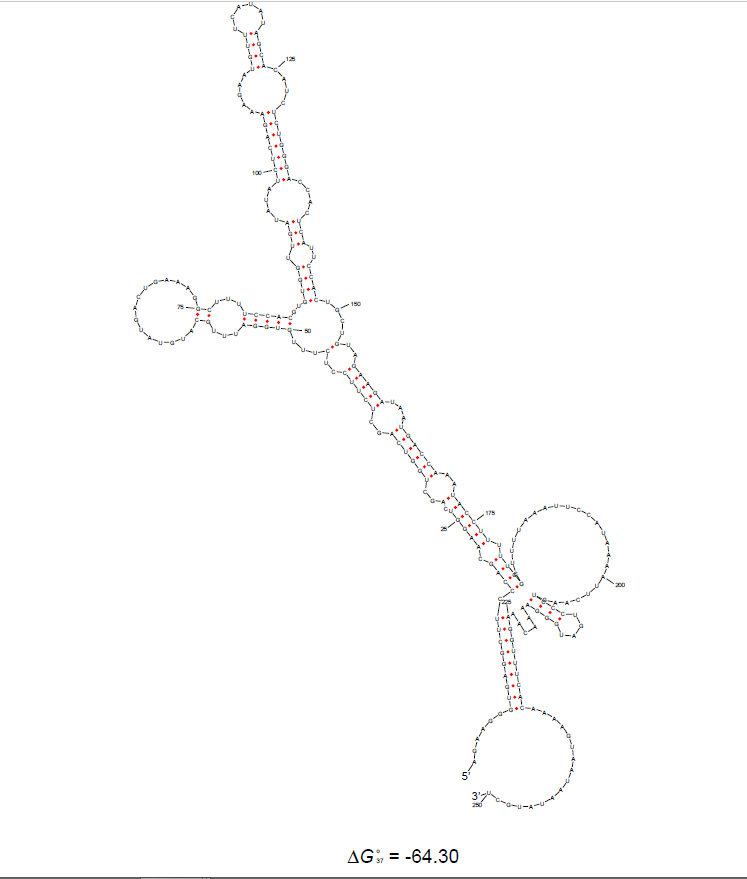 | 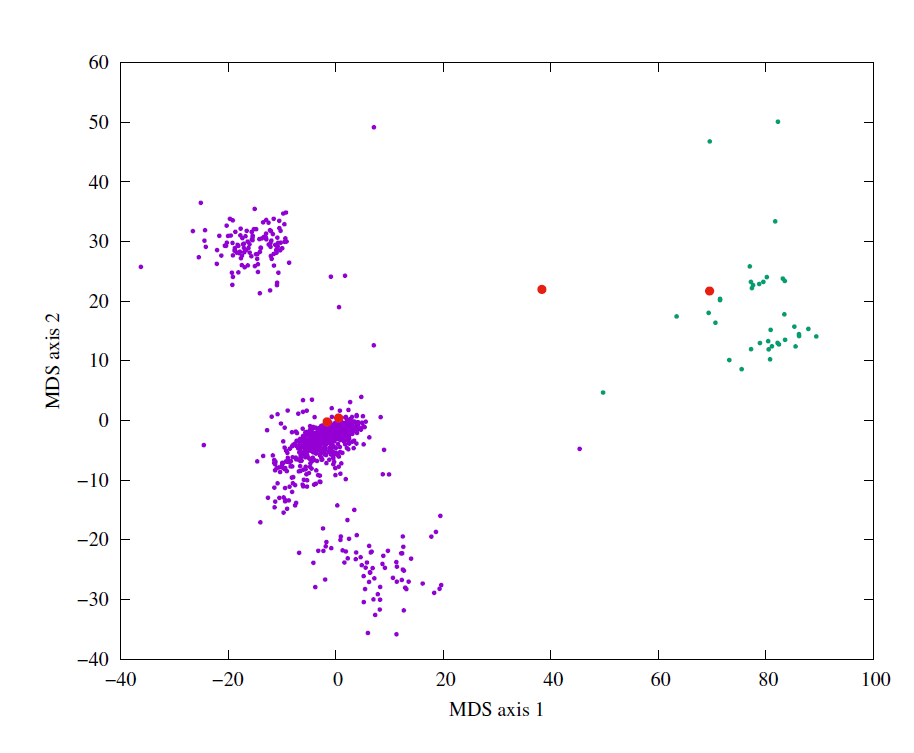 |
|  | C | 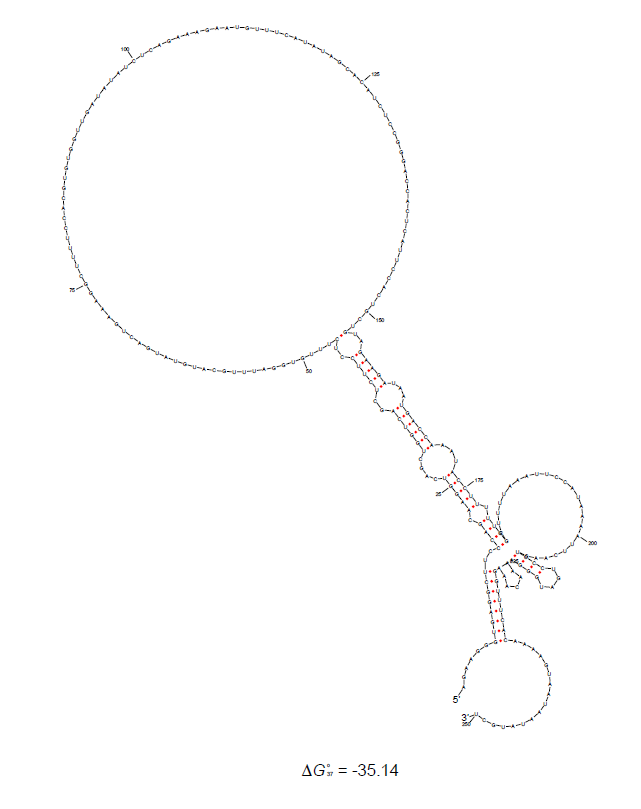 | 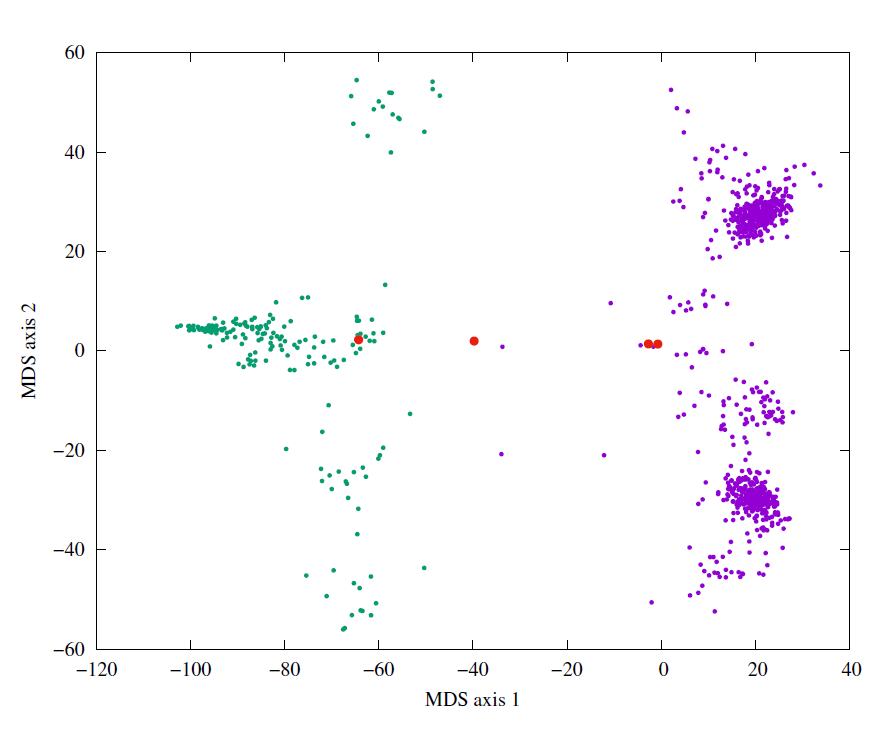 |
| **rs1333045** | T | 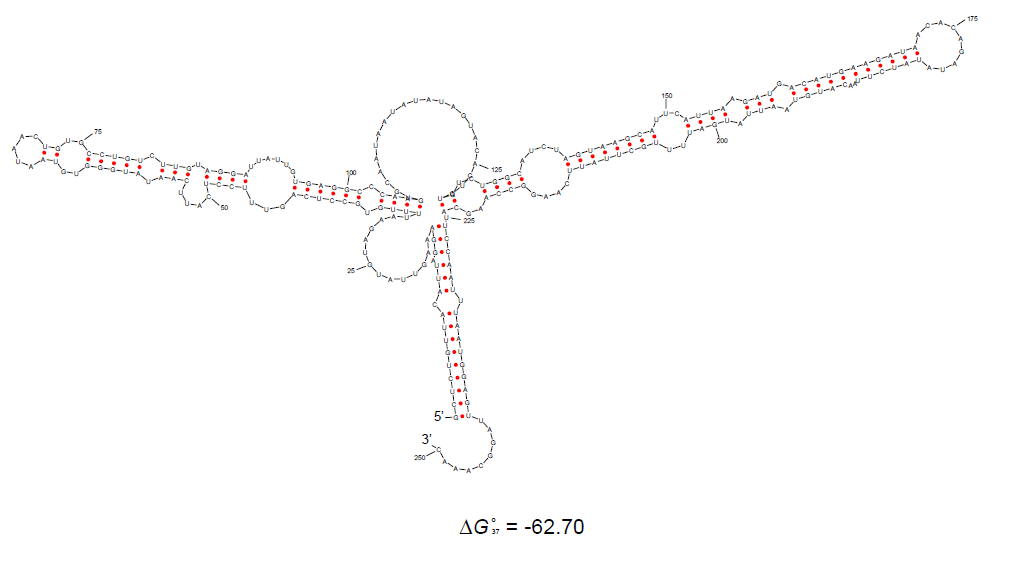 | 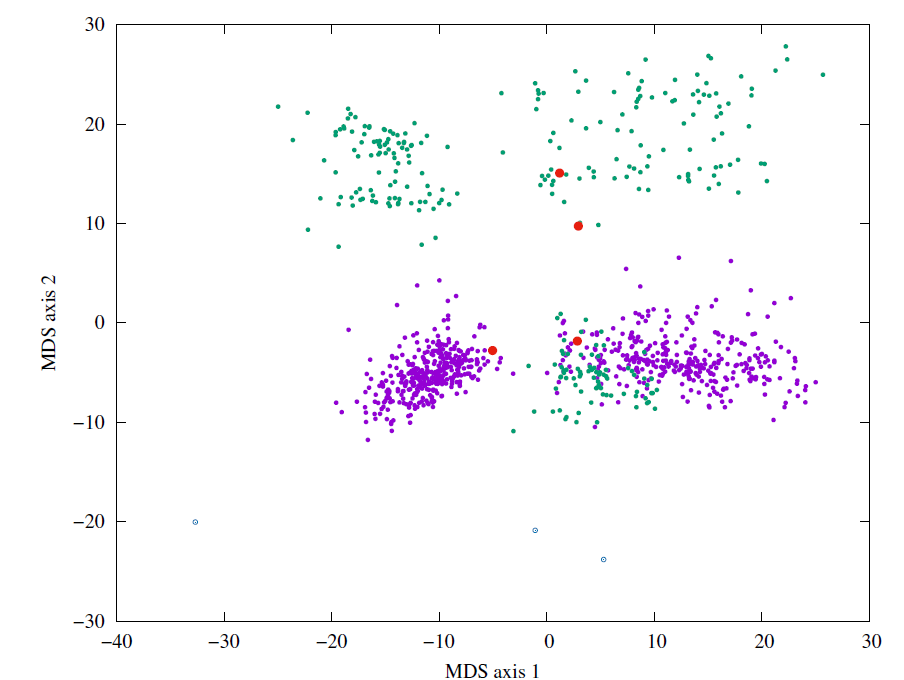 |
|  | C | 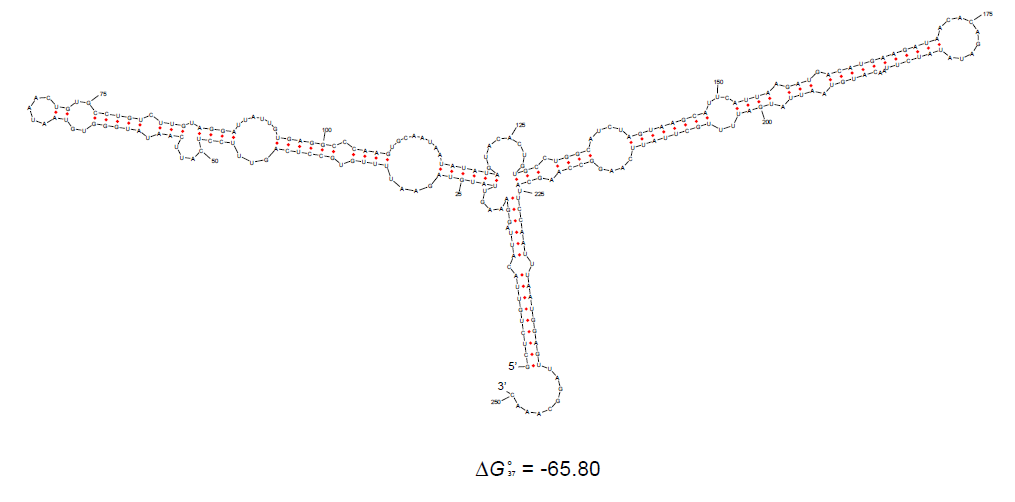 | 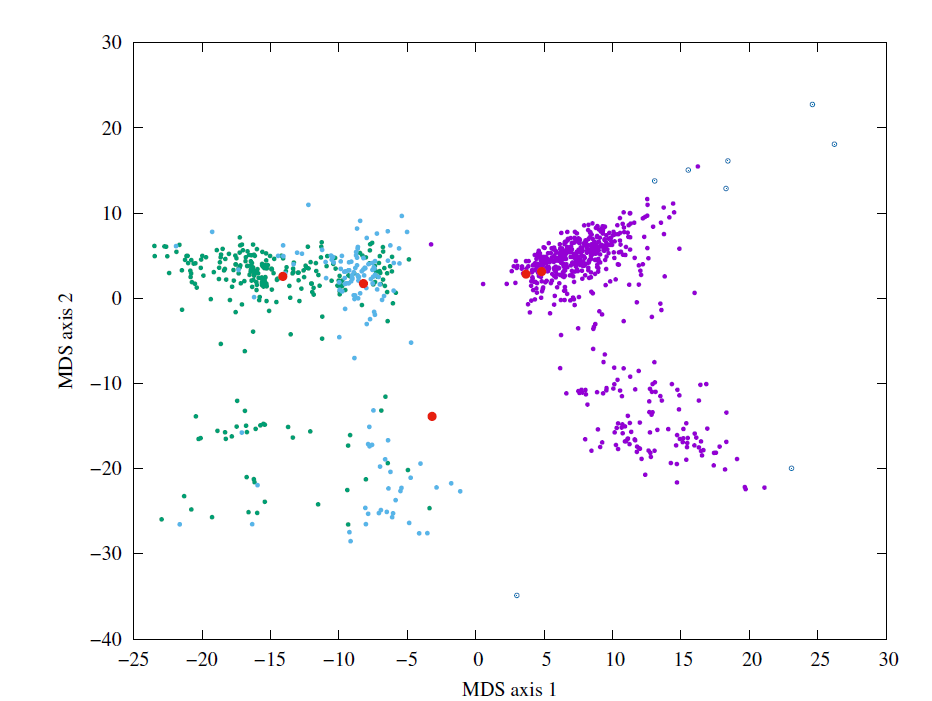 |

**Table S5. Analyzes of minimum free energies (MFE) for *IKZF3* mRNA.**

| **SNP** | **miRNA** | **Allele** | **miRNA-RNA structure** | **Allele** | **miRNA-RNA structure** |
| --- | --- | --- | --- | --- | --- |
| **rs907091** | *hsa-miR-LET7-a-2* | T | 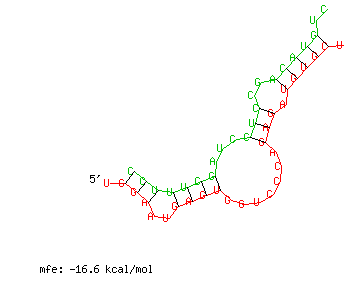 | C | 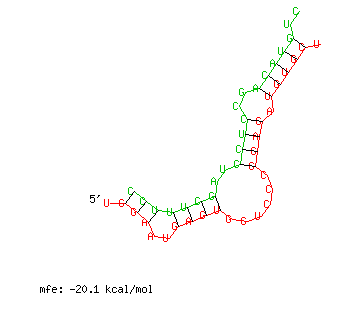 |
|  | *hsa-miR-1266* | T | 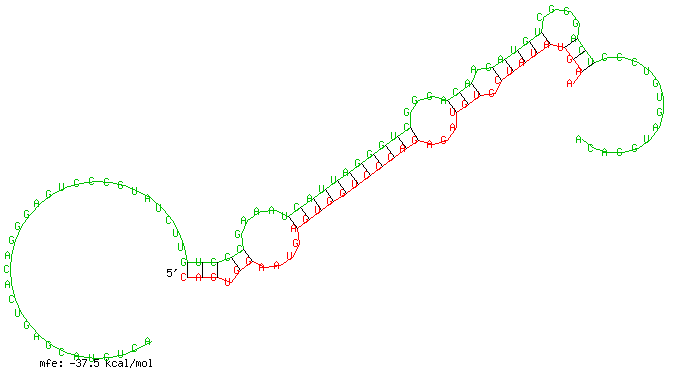 | C | 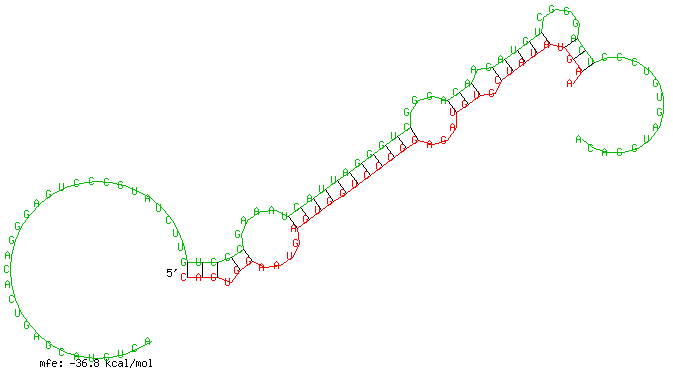 |
|  | *hsa-miR-211-3p* | T | 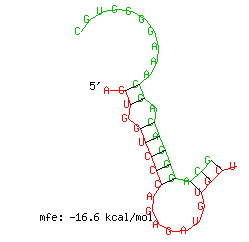 | C | 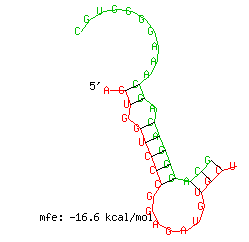 |
|  | *hsa-miR-3144-5p* | T | 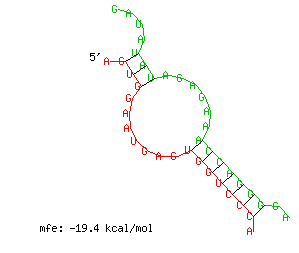 | C | 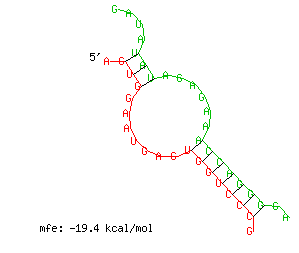 |
|  | *hsa-miR-3191-5p* | T | 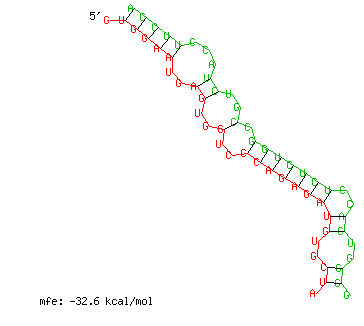 | C | 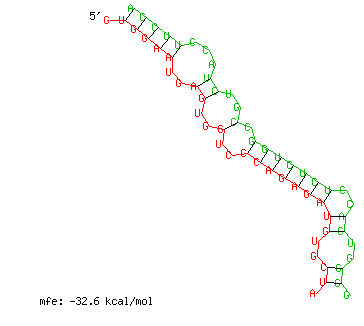 |
|  | *hsa-miR-326* | T | 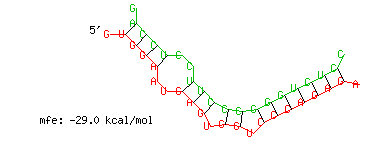 | C | 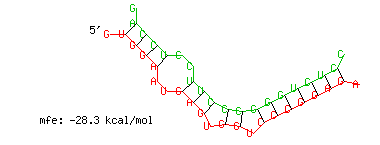 |
|  | *hsa-miR-330-5p* | T | 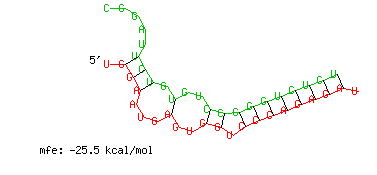 | C | 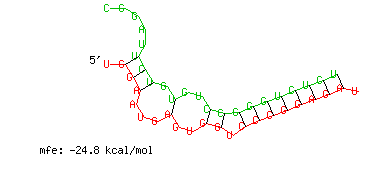 |
|  | \|  \| *hsa-mi-4314* \|  \| \| --- \| --- \| --- \| | T | 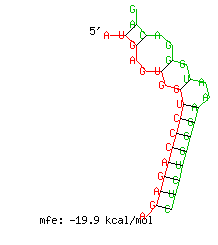 | C | 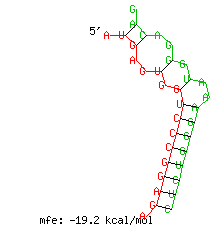 |
|  | *hsa-miR-4497* | T | 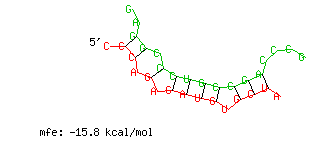 | C | 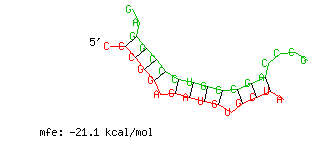 |
|  | *hsa-miR-4518* | T | 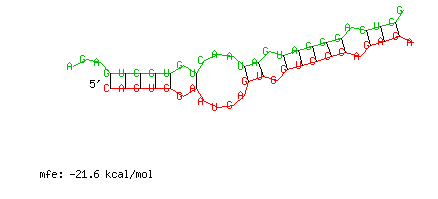 | C | 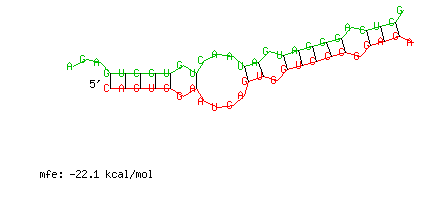 |

**MicroRNAs interaction in the presence of rs907091 alleles was assessed using RNAhybride.**
